# Evaluation of the Effect of Pressure-Controlled Ventilation-Volume Guaranteed Mode vs. Volume-Controlled Ventilation Mode on Atelectasis in Patients Undergoing Laparoscopic Surgery: A Randomized Controlled Clinical Trial

**DOI:** 10.3390/medicina59101783

**Published:** 2023-10-07

**Authors:** Ayse Zeynep Turan Civraz, Ayten Saracoglu, Kemal Tolga Saracoglu

**Affiliations:** 1 Department of Anesthesiology and Reanimation, Kocaeli City Hospital, Kocaeli 41060, Turkey; 2 College of Medicine, Qatar University, Doha P.O. Box 2713, Qatar; anesthesiayten@gmail.com (A.S.); saracoglukt@gmail.com (K.T.S.); 3 Department of Anesthesiology, ICU and Perioperative Medicine, Aisha Bint Hamad Hospital, Hamad Medical Corporation, Doha P.O. Box 3050, Qatar; 4 Anesthesiology Section, Hazm Mebaireek General Hospital, Hamad Medical Corporation, Doha P.O. Box 3050, Qatar

**Keywords:** lung ultrasonography, atelectasis, laparoscopic surgery, pressure-controlled ventilation, volume-controlled ventilation

## Abstract

*Background and Objectives*: Laparoscopic surgery, which results in less bleeding, less postoperative pain, and better cosmetic results, may affect the lung dynamics via the pneumoperitoneum. After laparoscopic surgery, atelectasis develops. The primary aim of the present study is to demonstrate the effects of two different ventilation modes on the development of atelectasis using lung ultrasound, and the secondary outcomes include the plateau pressure, peak inspiratory pressure, and compliance differences between the groups. *Materials and Methods*: In this study, 62 participants aged 18–75 years undergoing laparoscopic cholecystectomy were enrolled. The patients were randomly assigned into two groups: the volume-controlled ventilation (VCV) group (group V) or the pressure-controlled-volume guaranteed ventilation (PCV-VG) group (group PV). The lung ultrasound score (LUS) was obtained thrice: prior to induction (T1), upon the patient’s initial arrival in the recovery room (T2), and just before departing the recovery unit (T3). The hemodynamic data and mechanical ventilation parameters were recorded at different times intraoperatively. *Results*: The LUS score was similar between the groups at all the times. The change in the LUS score of the right lower anterior chest was statistically higher in the VCV group than the PCV group. The peak inspiratory pressure (PIP) was found to be statistically higher in the V group than the PV group five minutes after induction (T_5_) (20.84 ± 4.32 *p* = 0.021). The plateau pressure was found to be higher in the V group than the PV group at all times (after induction (T_ind_) 17.29 ± 5.53 *p* = 0.004, (T_5_) 17.77 ± 4.89 *p* = 0.001, after pneumoperitoneum (T_PP_) 19.71 ± 4.28 *p* = 0.002). Compliance was found to be statistically higher in the PV group than the V group at all times ((T_ind_) 48.87 ± 15.37 *p* = 0.011, (T_5_) 47.94 ± 13.71 *p* = 0.043, (T_PP_) 35.65 ± 6.90 *p* = 0.004). Before and after the pneumoperitoneum, the compliance was determined to be lower in the V group than the PV group, respectively (40.68 ± 13.91 *p* = 0.043, 30.77 ± 5.73 *p* = 0.004). *Conclusions*: LUS score was similar between groups at all times. The PCV-VG mode was superior to the VCV mode in providing optimal ventilatory pressures and maintaining high dynamic compliance in patients undergoing laparoscopic abdominal surgery.

## 1. Introduction

Optimizing intraoperative mechanical ventilation plays an important role in the reduction in postoperative pulmonary complications [1]. Laparoscopic surgery, the prevalence of which has been increased in recent years, causes less bleeding, less postoperative pain, and provides a shorter hospital stay and better cosmetic results compared to the open procedure. However, although it is less invasive, the lung dynamics may be affected by the resulting pneumoperitoneum. Atelectasis develops after laparoscopic surgeries with an incidence of 2.4% reported in the PERISCOPE study, one of the most comprehensive studies addressing postoperative complications [2]. A previous study reported a 21–46% decrease in the FVC, 20–46% in the FEV1, and 12–32% in the FEF 25–75% 24 h after laparoscopic cholecystectomy as compared to preoperative values, as well as an 8% decrease in the total lung capacity [3]. The reasons for this include general anesthesia, paralysis with muscle relaxant agents, a cephalad shift of the diaphragm due to the decrease in lung volumes with increased intra-abdominal pressure as a result of pneumoperitoneum, absorption atelectasis due to the high FiO_2_ applied in the perioperative period, decreased respiratory compliance, negative pressure during extubation, and lack of lung protective ventilation. Increasing atelectasis augments the pulmonary shunts by decreasing the number of ventilated alveoli, which leads to ventilation perfusion mismatch. The resulting atelectasis is blamed for elevated postoperative mortality and morbidity and the high costs and labor loss due to extended hospital stays [4,5].

Although the use of volume-controlled ventilation (VCV) guarantees the target minute volume for the patient, any change in compliance or resistance can lead to high peak inspiratory pressures with constant flow, causing both barotrauma and changes in pulmonary gas distribution. On the other hand, the application of high-frequency low-tidal-volume ventilation to prevent this may cause atelectasis and deteriorate arterial oxygenation [6]. However, there is a consensus that low-volume, low-pressure, and high-frequency ventilation is lung-protective ventilation and superior to the high-volume ventilation; atelectasis is linked to decreased compliance in the lower respiratory system and lungs. This is due to the fact that the closure of alveoli and small airways coincides with the low-volume section of the pressure-volume curve, resulting in a smaller change in lung volumes in response to applied pressures, i.e., low compliance [7].

Pressure-controlled volume-guaranteed ventilation (PCV-VG) is a ventilation mode in which constant pressure is applied with a decelerating flow. PCV-VG is based on the principle of allowing the target tidal volume to be inhaled by the patient at each mandatory breath without increasing the airway pressure. It is made possible by automatically changing the ventilation parameters, i.e., initially delivering a high inspiratory flow and then providing a decreasing flow. In this way, it may be possible to prevent the ventilation–perfusion mismatch by ensuring a more homogeneous distribution of ventilation and preventing the increase in peak inspiratory pressure. In other words, the PCV-VG is a combination of the advantages of both pressure-controlled ventilation (PCV) and VCV and enables the application of target minute ventilation without causing barotrauma [8,9]. Song et al. [10] compared the PCV-VG and VCV modes in patients undergoing single lung ventilation and found that although airway pressures were lower in the PV group, the arterial oxygen pressures were similar in patients receiving ventilation in both modes.

Lung ultrasonography (LUS) has begun to be used in clinical settings as a new tool for the detection of both acute and chronic pathologies during the perioperative and intensive care follow-up thanks to its various features, including use at the bedside, giving rapid results, being noninvasive and radiation-free, and not requiring patients to be transported [11]. There are studies suggesting that atelectasis can be prevented with the use of LUS during laparoscopic surgery. Lung ultrasonography enables medical professionals to image patients throughout an operation, even while it is still in progress [12]. In patients who are under anesthesia and scheduled for surgery, lung ultrasonography can detect intraoperative atelectasis, and the LUS score is correlated with perioperative oxygenation impairment [13]. It permits careful observation of the regional lung aeration changes brought on by prone positioning, fluid loading, positive end-expiratory pressure, and drainage of pleural effusions.

The visualization pattern of atelectasis resembles lung consolidation, and loss of lung sliding and B-lines are considered to be the typical findings related to the disorder [14].

Different protective ventilation strategies have been used to reduce the development of atelectasis after laparoscopic surgery, and researchers have attempted to determine the effect of VCV and PCV-VG on the development of atelectasis based on the lung dynamics, hemodynamic data, and blood gas measurements. However, it is thought that with the use of lung ultrasonography, atelectasis can be detected at the bedside in the early postoperative period, and thus postoperative mortality and morbidity can be reduced with early treatment. Nevertheless, there is no study in the literature designed to use lung ultrasonography for this purpose.

In the present study, using lung ultrasonography, we aimed to demonstrate the effect of the volume-controlled ventilation and pressure-controlled volume-guaranteed ventilation on atelectasis developing in the perioperative period in patients undergoing laparoscopic surgery.

## 2. Materials and Methods

### 2.1. Study Design and Study Participants

This prospective, single-centered, and randomized controlled trial was conducted in Derince Training and Research Hospital from September 2018 to September 2019. The study was conducted in accordance with the Declaration of Helsinki and approved by the Kocaeli University Ethical Committee of Clinical Research (KOU KIA 2018/260). The study was registered on clinicaltrials.gov (trial registration number: 02/08/2018 NCT03614845). Before randomization, written informed consent was obtained from all participants during preoperative evaluation. The study adhered to the reporting standards established by the Consolidated Standards of Reporting Trials (CONSORT).

### 2.2. Selection and Exclusion Criteria

**Figure 1:**
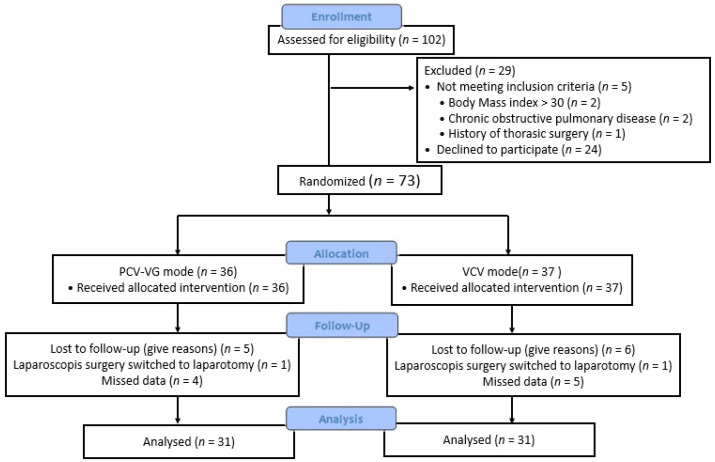
Flow diagram of the study.

Eligible patients were American Society of Anesthesiologists (ASA) I-II patients aged between 18 and 75 years who were scheduled for laparoscopic cholecystectomy. There are patient-induced, surgical-induced, and anesthesia-induced risk factors in the development of perioperative atelectasis [15]; therefore, patients with patient-related risk factors for developing atelectasis, including a BMI over 30, chronic heart or lung disease, OSAS leading to inadequate ventilation postoperatively, thoracic surgery or trauma resulting in confusing lung ultrasound images, and ASA classification 3–4, were excluded from this study (Figure 1).

### 2.3. Randomization and Blinding

The patients were divided into two groups with the closed envelope method to undergo either volume-controlled ventilation (Group V) or pressure-controlled volume-guaranteed ventilation (Group PV). Opaque envelopes with sequential numbers were created by an individual who was not involved in the study. These envelopes were opened by an independent party who was also not involved in the study or the preparation of the envelopes. Intraoperative hemodynamic data and ventilation parameters, predetermined in the study protocol, were recorded on patient monitors and subsequently transcribed onto paper case report forms by an anesthesia assistant who was blinded to the protocol and hypothesis of the study and group assignments. Lung ultrasonography was performed by an anesthesiologist who was blind to the study groups and experienced in lung ultrasonography.

### 2.4. Anesthesia and Surgical Procedure

In the operating room, the patients were monitored in terms of an electrocardiogram (EKG), peripheral oxygen saturation, noninvasive blood pressure, heart rate, and body temperature. Following the induction of anesthesia, the end-tidal CO_2_ (ETCO_2_) was monitored.

For anesthesia induction, patients were given an IV bolus dose of propofol 2 mg/kg, fentanyl 2–3 μg/kg, and rocuronium bromide 0.6 mg/kg. Then, they were intubated with a cuffed endotracheal tube. While 7–7.5 mm tubes were used for women, 8–8.5 mm tubes were used for men. IV remifentanil infusion (0.2 μg/kg/min) and Sevoflurane 2% in a 1:1 air:oxygen mixture were used to maintain anesthesia. Low-flow anesthesia was used with a fresh gas flow rate of 1 L/min; an inspired oxygen concentration (FIO2) of 0.5 was provided along with air. The oxygen saturation was targeted above 93% throughout the operation [16]. The depth of anesthesia was monitored with a Bispectral Index (BIS, Philips Healthcare, Andover, MA, USA) monitor throughout the operation, and the anesthesia was maintained at a BIS value of between 40 and 60. In the perioperative period, the patients were ventilated using the Datex-Ohmeda-Avance CS2 Anesthesia Device. The ventilation parameters were set as follows: 7 mL/kg (predicted body weight) tidal volume, inspiration/expiration (I/E) ratio 1:2, positive end-expiratory pressure (PEEP): 5 cmH_2_0, and respiratory rate ETCO_2_: 35 ± 2 mmHg. The intraperitoneal pressure was kept constant at 12 ± 2 mmHg in both groups.

In the perioperative period, the peak and plateau inspiratory pressures (IP), dynamic compliance, respiratory rate, exhaled tidal volume and ETCO_2_, and oxygen saturation (SpO_2_) were recorded right after induction (T_ind_), 5 min after induction (T_5_), and after pneumoperitoneum (T_PP_). We recorded, as hemodynamic data, the blood pressure, heart rate, and body temperature; as demographic data, age, sex, BMI, and ASA score; and as surgical data, the duration of anesthesia and surgery, the length of pneumoperitoneum, and the volume of administered fluid. Perioperative desaturation was defined as a peripheral oxygen saturation below 94%. In that case, the FiO_2_ was increased to 100%, and then oxygen titration was set to the baseline level.

The patients’ perioperative fluid requirement was met through 5 mL/kg/hr IV infusion of crystalloid solutions. In case of perioperative bleeding, the first 500 mL was compensated with colloid liquids. Continuous hemorrhages were replaced with blood after estimating the blood volume patients lost. At the end of surgery, CO_2_ insufflation was stopped, and the patients were taken to the recovery room after the neuromuscular block was reversed with sugammadex. After analgesia was achieved, lung ultrasonography was performed when the Aldrete score was >9. Patients were followed up for other major postoperative complications.

### 2.5. Lung Ultrasonography

Lung ultrasonography was performed with a Mindray M5 Mobile Trolley^®^ (Shenzhen Mindray Bio-Medical Electronics Co., Ltd., Hamburg, Germany) branded ultrasound machine using a convex probe by an anesthesiologist who was blind to the study protocol. Measurements were conducted prior to anesthesia induction (T1), as soon as the patient was taken to the recovery unit (T2), and just before the patient was transferred to the ward (T3), on the basis that the focal zone on the pleural line was determined.

**Figure 2:**
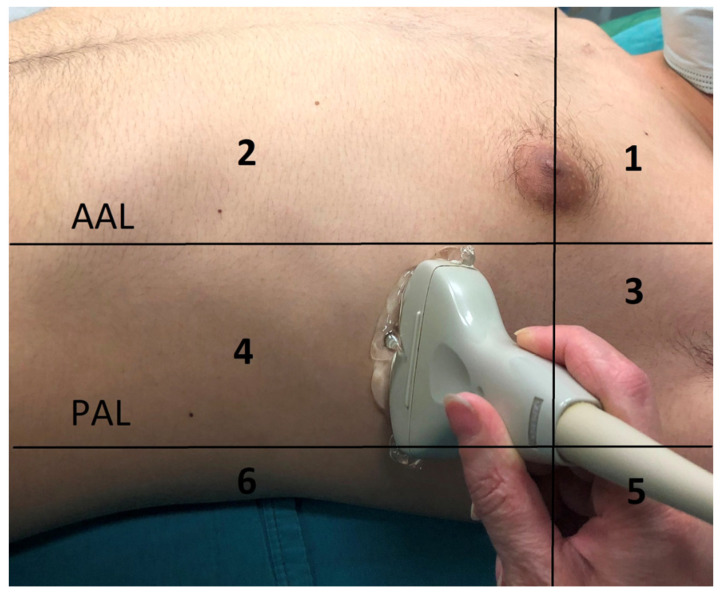
Divided regions of thorax for lung ultrasonography. AAL: anterior axillary line; PAL: posterior axillary line. 1. anterosuperior region, 2. anteroinferior region, 3. laterosuperior region, 4. lateroinferior region, 5. posterosuperior region, 6. posteroinferior region.

The thorax was divided into 12 quadrants according to the anterior and posterior axillary line in both the right and left lung areas; anterior, lateral, and posterior zones were identified, and the upper and lower regions (R1, R2 … R6; L1, L2 … L3) of these zones were also visualized. (Figure 2) Aeration loss was determined considering the Modified LUS score. Each of these 12 quadrants scored between 0 and 3, and the total score (0–36) revealed the degree of loss of aeration [12].

During laparoscopic cholecystectomy procedures, patients are placed in the Trendelenburg position in order to enhance surgical vision. However, as the surgical area is located in the upper right quadrant, the most critical anatomical area for surgical manipulation is the area under the right diaphragm, which fits under the right lung. For these reasons, we sought to conduct a detailed analysis of the LUS score in each region.

### 2.6. Outcome Measurement of the Study

Outcomes measures: the primary outcome of the present study is the difference in the LUS score between the two groups in postoperative periods T2 and T3; secondary outcomes include the plateau pressure, peak inspiratory pressure, and compliance differences between the groups at (T_ind_), (T_5_), and (T_PP_).

### 2.7. Statistical Analysis

The SPSS 22.0 program was used for analysis. We used the mean, standard deviation, median, minimum, maximum, frequency, and ratio values for descriptive statistics. The distribution of variables was measured with the Kolmogorov–Smirnov test. As we had a small sample size, it was important to determine the distribution of the variable in order to choose an appropriate statistical method, so the Shapiro–Wilk test was performed and showed non-normality, and the results were consistent with the Kolmogorov–Smirnov test. The Kruskal–Wallis and the Mann–Whitney tests were used in the analysis of quantitative independent data. Dependent quantitative data were analyzed with the Wilcoxon test. Quantitative independent data were analyzed using the chi-square test, but when the conditions for the chi-square test were not met, we used the Fisher test. For the analysis of repeated data, the repeated measures ANOVA was used. *p* < 0.05 was considered statistically significant. If feasible, the Tukey HSD test was used for post hoc analyses.

The calculation of the sample size was based on a previous study [9], and the mean and standard deviation of the peak inspiratory pressure value of the two different ventilation modes was used. Using an alpha level of 0.05 and a desired power of 80%, we calculated that a total of 24 participants was required in each group to demonstrate a statistically significant difference. We recruited an additional 20% to account for dropouts. A *p*-value of less than 0.05 was considered to be statistically significant. The Minitab software program was used to calculate sample size and power. Power = 0.89 in our study; with 62 patients in our study, the sample size of our study was sufficient, and our study had a very high reliability.

## 3. Results

### 3.1. Analyses of Demographics and Surgical Data

#### 3.1.1. Demographic and Surgical Characteristics

In total, 102 patients were followed up for this study. Twenty-four patients declined to participate in the study, and five patients did not meet the criteria for inclusion. Nine patients were excluded from the study due to missing data, and two patients had their surgeries switched from laparoscopy to laparotomy. In conclusion, 62 patients’ data were analyzed.

The patients were aged from 19 to 75 years of age (48.66 ± 15.15 years). While 31 patients received the PCV-VG mode, the other 31 patients received the VCV mode. Of the study participants, 46 (74.2%) were female and 16 (25.8%) were male.

The demographic and surgical data were statistically similar between the two groups of the patients participating in the study (Table 1)

#### 3.1.2. Analyses of Hemodynamic Parameters

The mean arterial pressure (MAP) and heart rate during the perioperative period were found to be similar. The ventilator parameters of the SPO_2_, ETCO_2_, and tidal volume were statistically similar between the groups. Neither intraoperative nor postoperative major complication was observed in the patients included in the study. The mean perioperative fluid infusion was 700 mL and similar between the two groups. Infusion of 0.9% normal saline was used as the maintenance fluid. No patient suffered from bleeding or required a blood transfusion.

### 3.2. Analyses of LUS Scores

The total LUS scores were statistically significantly similar between the groups (Table 2).

However, when the change in LUS measurements was investigated by region, the postoperative L2, L4, L5, L6, R2, R3, R4, R5, and R6 values of the patients in the PV group were statistically significantly higher compared to the preoperative values (*p* < 0.05). The postoperative L1, L3, and R1 values were found to be higher than the preoperative L1, L3, and R1 values, but the difference was not statistically significant (Table S1).

In the V group, on the other hand, the postoperative L1, L2, L3, L5, L6, R1, R2, R3, R4, R5, and R6 values were statistically significantly higher than the preoperative L1, L2, L3, L5, L6, R1, R2, R3, R4, R5, and R6 values (*p* < 0.05). The postoperative L4 value was greater than the preoperative L4 value, but there was no statistically significant difference (Table S2).

The change in the postoperative LUS score in the R2 region was statistically significantly higher in the V group compared to the PV group (*p* < 0.05) (Table 3). However, there was no statistically significant difference between the groups regarding the rate of change in other regions.

There was no significant difference between the two groups in the mean intraabdominal pressure, and the maximum intra-abdominal pressure was kept around 15 mmHg.

### 3.3. Analyses of Ventilator Parameters

The peak inspiratory pressure value prior to the pneumoperitoneum was statistically significantly higher in the V group as compared to the PV group (*p* < 0.05). The other values were statistically significantly similar between the groups.

The plateau pressure measurements were statistically significantly higher in the V group as compared to the PV group (*p* < 0.05).

The compliance measurements were statistically significantly lower in the V group as compared to the PV group (*p* < 0.05). Moreover, it was observed that the measurement before pneumoperitoneum was 15% lower in the V group compared to the PV group, and the time measurement after pneumoperitoneum was 14% lower in the V group compared to the PV group (Table 4).

The compliance value after pneumoperitoneum was statistically significantly lower compared to the other time measures (*p* < 0.05). The peak inspiratory pressure and plateau pressure values after pneumoperitoneum were statistically significantly greater compared to the other time periods (*p* < 0.05) (Table S3).

## 4. Discussion

Using lung ultrasound, we found that aeration loss occurs during laparoscopic cholecystectomy in this randomized controlled trial. The ventilation modes, VCV and PCV-VG, did not influence the development and amount of atelectasis. LUS scores showed less aeration loss in the PCV-VG group only in the right region. The PCV-VG ventilation mode was associated with better lung mechanics in the patients.

In our study, the results of the analysis of the mechanical ventilation parameters may be attributed to some advantages that the PCV-VG mode provides over the VCV. The PCV-VG mode delivers the preset tidal volume with a decelerating flow at the lowest possible pressure. When using the pressure-controlled ventilation mode, the required tidal volume is provided to the patient performing the compliance changes required to achieve a consistent tidal volume. The PCV-VG offers the benefits of PCV with better oxygenation and the security of consistent tidal volume. Due to all these reasons, the PCV-VG is useful for any surgical procedure including laparoscopy, where the patient’s compliance is expected to change perioperatively. As compared to volume-controlled ventilation, it provides better oxygenation at lower peak inspiratory pressures and higher mean airway pressures [17].

Volume-controlled ventilation has been the traditional mode of controlled ventilation in anesthesia. In VCV, the ventilator delivers the preset tidal volume with a constant flow at the preset respiratory rate during a preset inspiratory time. It has been the preferred mode of mechanical ventilation in anesthesia applications due to its ability to control Minute Volume. However, the VCV uses a constant flow that may cause high peak pressures and thus expose the patient to the risk of barotrauma [18]. In our study, we detected a significant increase in the inspiratory pressures of patients who were applied VCV, but that increase was not at a level to cause barotrauma. Gas distribution in the lungs may not be optimal during VCV because the alveoli may not effectively fill until the end of the inspiratory time, leaving less time for gas exchange. The combination of constant flow and high resistance may generate very high inspiratory pressures.

Similarly in the study of Assad et al., the PCV-VG mode was determined to be superior to the VCV mode with a significantly lower peak inspiratory pressure and greater dynamic compliance than the VCV mode [9]. Cadi et al. [19] compared PCV with VCV in laparoscopic obesity surgery and revealed the advantages of PCV in ventilation parameters. After that, Toker et al. [20] compared VCV and PCV-VG modes in laparoscopic gynecologic surgery. In this study, Toker et al. reported an improvement in oxygenation as well as in the PCV-VG ventilation parameters. The common point of the two studies is that they were conducted on obese patients. Unlike these two studies, we included patients with a BMI of <30 kg/m^2^ in our study and indicated that the PCV-VG mode is useful in non-obese patients as well.

Another study on laparoscopic gynecological surgeries drew a comparison between VCV and PCV and reported a significant increase in the peak airway pressure, compliance, plateau pressure, and airway resistance when VCV was used [21]. Changes in compliance and resistance affect the tidal volume administered in most cases. Similarly, there was a decrease in compliance in patients receiving VCV in our study. In pressure-controlled modes, the peak inspiratory pressure is guaranteed by the ventilator; thus, the preset pressure limits are not exceeded. In this way, it becomes possible to minimize the risk of alveolar overdistention and barotrauma.

One of the unique features of our study is that we evaluated aeration loss with postoperative LUS scores. When the LUS scores were evaluated, it was observed that all patients developed aeration loss following laparoscopic surgery with elevated LUS scores. The increase in the incidence of aeration loss in these patients was regardless of the perioperative mechanical ventilation parameters. However, it is worthy of note that the change in the postoperative LUS score inferoanterior zone of the right lung was significantly greater in the V group compared to the PV group. Another advantage besides effective ventilation was that atelectasis complication did not occur.

In addition to intraoperative techniques to prevent atelectasis in abdominal surgeries, it would be pertinent to discuss several postoperative strategies. These include the use of noninvasive ventilator support or high-flow nasal cannula in high-risk patients such as those with a BMI > 30, respiratory and cardiac illnesses, prolonged surgery duration, and major surgery. Both these strategies can be employed as prophylactics, as well as therapeutics, in the postoperative period.

Strategies to prevent atelectasis in the postoperative period include early mobilization, respiratory physiotherapy that expands the lungs, non-opioid analgesia that is effective, oxygen therapy, mucolytics, eliminating causes that may cause external compression of the thorax, reducing abdominal pressure, evacuating the stomach with a nasogastric tube, and draining any pleural effusion that may be present in the thorax [15].

### Limitations

The major limitation of the present study is that while the sample size and expressive power of our study are sufficient, it is important to note that this study was conducted on a singular type of surgery and a specific patient group. Further studies will need to include larger series and more diverse patient groups; for example, obese patients, patients with severe cardiopulmonary problems, the pediatric age group, and patients undergoing different types of surgery. Another limitation is that ultrasonography was used for the diagnosis of atelectasis only in the postoperative period, whereas frequent intraoperative measurements of LUS score may detect atelectasis accurately.

## 5. Conclusions

During laparoscopic cholecystectomy, atelectasis can occur due to the increase in intra-abdominal pressure and the application of the Trendelenburg position to the patient. Although the PCV-VG mode is superior to the VCV mode in providing compliance and preventing peak and inspiratory pressure increases, this was not reflected in the LUS scores in our study. At this point, we believe that further research is needed.

## Figures and Tables

**Table 1:** Demographic and surgical data of the groups.

	Group PV	Group V	*p*
	(*n* = 31) mean ± SD (median)	(*n* = 31) mean ± SD (median)	
Sex		24 (77.45%)	0.772 ᵏ
Female	22 (71.0%)		
Male	9 (29.0%)	7 (22.6%)	
Age (years)	49.48 ± 14.83	47.84 ± 15.67	0.673 ᶜ
BMI ^1^	29.03 ± 4.99	28.45 ± 4.99	0.648 ᶜ
Anesthesia duration (min)	58.84 ± 13.24	63.61 ± 17.16	0.225 ᶜ
Duration of surgery (min)	47.45 ± 13.26 (45)	49.90 ± 16.03 (45)	0.665 ᵈ
Duration of pneumoperitoneum (min)	38.71 ± 13.58 (40)	40.94 ± 15.17 (40)	0.744 ᵈ
Length of stay at postoperative ward (min)	27.58 ± 3.62 (30)	28.06 ± 3.37 (30)	0.579 ᵈ
Aldrete Score	9.61 ± 0.49 (10)	9.71 ± 0.46 (10)	0.425 ᵈ

^1^ BMI: body mass index. ᵏ Chi-square test: values are given as frequency (percentage); ᵈ Mann–Whitney U test: values are given as mean ± standard deviation (median); ᶜ independent samples T test: values are given as mean ± standard deviation.

**Table 2:** LUS scores of the patients.

	Group PV	Group V	*p*
	(*n* = 31) mean ± SD (median)	(*n* = 31) mean ± SD (median)	
Preoperative (T1)	4.94 ± 3.95 (4)	3.9 ± 4.22 (2)	0.231 ᵈ
Postoperative 1 (T2)	10.23 ± 5.25 (12)	10.71 ± 4.58 (11)	0.740 ᵈ
Postoperative 2 (T3)	8.48 ± 4.60	8.81 ± 5.02	0.793 ^c^

ᵈ Mann–Whitney U test: values are given as mean ± standard deviation (median); ᶜ independent samples *t* test: values are given as mean ± standard deviation.

**Table 3:** Comparison of the LUS scores of the right regions between the groups.

	PCV	VCV	*p*
	(*n* = 31) mean ± SD (median)	(*n* = 31) mean ± SD (median)	
R1 preoperative	0.39 ± 0.495 (0.0)	0.23 ± 0.425 (0.0)	0.172
R1 postoperative	0.65 ± 0.61 (1.0)	0.58 ± 0.67 (0.0)	0.588
*p*	0.92	0.024 *	
R2 preoperative	0.71 ± 0.59 (1.0)	0.39 ± 0.615 (0.0)	0.022 *
R2 postoperative	1.26 ± 0.63 (1.0)	1.58 ± 0.564 (2.0)	0.037 *
*p*	0.001 *	0.001 *	
R3 preoperative	0.55 ± 0.51 (1.0)	0.39 ± 0.56 (0.0)	0.170
R3 postoperative	0.90 ± 0.70 (1.0)	0.94 ± 0.57 (1.0)	0.804
*p*	0.043 *	0.001 *	
R4 preoperative	0.87 ± 0.72 (1.0)	0.74 ± 0.575 (1.0)	0.508
R4 postoperative	1.39 ± 0.715 (2.0)	1.55 ± 0.77 (2.0)	0.4330
*p*	0.007 *	0.001 *	
R5 preoperative	0.10 ± 0.30 (0.0)	0.03 ± 0.18 (0.0)	0.305
R5 postoperative	0.52 ± 0.57 (0.0)	0.71 ± 0.59 (1.0)	0.188
*p*	0.001 *	0.001 *	
R6 preoperative	0.19 ± 0.40 (0.0)	0.13 ± 0.43 (0.0)	0.316
R6 postoperative	0.97 ± 0.71 (1.0)	1.19 ± 0.70 (1.0)	0.208
*p*	0.007 *	0.001 *	

Mann–Whitney U test: values are given as mean ± standard deviation (median); * *p* < 0.05: statistically significant difference between groups.

**Table 4:** Peak inspiratory pressures, plateau pressures, and compliance values during surgery.

		Group PV	Group V	*p*
		(*n* = 31)	(*n* = 31)	
		mean ± SD (median)	mean ± SD (median)	
Peak Inspiratory Pressure	(T_ind_) (mmHg)	17.94 ± 4.26 (17)	20.52 ± 5.74 (18)	0.087 ^d^
	(T_5_) (mmHg)	18.42 ± 3.67	20.84 ± 4.32	0.021 *^,c^
	(T_PP_) (mmHg)	21.26 ± 3.46	22.97 ± 3.84	0.071 ^c^
Plateau Pressure	(T_ind_) (cmH_2_O)	13.71 ± 3.53 (12)	17.29 ± 5.53 (16)	0.004 *^,d^
	(T_5_) (cmH_2_O)	14.10 ± 3.01	17.77 ± 4.89	0.001 *^,c^
	(T_PP_) (cmH_2_O)	16.61 ± 3.23	19.71 ± 4.28	0.002 *^,c^
Compliance	(T_ind_) (mL/cmH_2_O)	48.87 ± 15.37 (52)	42.10 ± 12.22 (41)	0.011 *^,d^
	(T_5_) (mL/cmH_2_O)	47.94 ± 13.71	40.68 ± 13.91	0.043 *^,c^
	(T_PP_) (mL/cmH_2_O)	35.65 ± 6.90	30.77 ± 5.73	0.004 *^,c^

^d^ Mann–Whitney U test: values are given as mean ± standard deviation (median); ^c^ independent samples T test: values are given as mean ± standard deviation; * *p* < 0.05: statistically significant difference between groups.

## Data Availability

The authors confirmed that the data supporting the findings of this study are available within the article and its Supplementary Materials. Raw data that support the findings of this study are available from the corresponding author, upon reasonable request.

## Supplementary Materials

The following supporting information can be downloaded at https://www.mdpi.com/article/10.3390/medicina59101783/s1: Table S1: Detailed Regional Comparison of the PCV in-group LUS Scores between Preoperative and Postoperative Measurements; Table S2: Detailed Regional Comparison of the VCV in-group LUS Scores between Preoperative and Postoperative Measurements; Table S3: Analyses of ventilator parameters at different timepoints.
