# Supplementary material for: Evaluation of the Effect of Pressure-Controlled Ventilation-Volume Guaranteed Mode vs. Volume-Controlled Ventilation Mode on Atelectasis in Patients Undergoing Laparoscopic Surgery: A Randomized Controlled Clinical Trial
Source: Medicina (Kaunas). 2023 Oct 7;59(10):1783. doi: 10.3390/medicina59101783 (PMC10607930; doi:10.3390/medicina59101783)
Supplement: Supplementary file 1 [file medicina-59-01783-s001.zip › medicina-2618223-supplementary.pdf]

**Tablo S3. Analyse of ventilator parameters at different timepoints**				
	T_{ind} (n=31)	T_5 (n=31)	T_{PP} (n=31)	p
PIP PCV	17,94±4,26	18,42±3,67	21,26±3,46	0.002*[a]
PIP VCV	20,52±5,74(18)	20,84±4,32(20)	22,97±3,84(24)	0.037*[b]
Plateau pressure PCV	13,71±3,53(12)	14,09±3,01(13)	16,61±3,23(16)	0.001*[b]
Plateau pressure VCV	17,29±4,89(16)	17,77±4,89(18)	19,71±4,28(19)	0.074[b]
Compliance PCV	48,87±15,37(52)	47,94±13,71(48)	35,64±6,90(35)	0.001*[b]
Compliance VCV	42,09±12,22	40,68±13,91	30,77±5,73	0.001*[a]

[a] One-Way ANOVA test: values are given as mean ± standard deviation

[b] Kruskal Wallis H test: values are given as mean ± standard deviation (median)

*p<0.05 statistically significant differecence among groups

**Tablo S1. Detailed Regional Comparison of PCV in-group LUS Scores between Preoperative and Postoperative Measurements**			
	Preoperative (n=31)	Postoperative (n=31)	p
L1	0,39±0,495(0,0)	0,61±0,615(1,0)	0.150
L2	0,48±0,57(0,0)	0,94±0,68(1,0)	0.008*
L3	0,48±0,57(0,0)	0,74±0,63(1,0)	0.100
L4	0,65±0,55(1,0)	1,06±0,68(1,0)	0.013*
L5	0,10±0,30(0,0)	0,39±0,495(0,0)	0.008*
L6	0,10±0,30(0,0)	0,65±0,486(1,0)	0.001*
R1	0,39±0,495(0,0)	0,65±0,61(1,0)	0.092
R2	0,71±0,59(1,0)	1,26±0,63(1,0)	0.001*
R3	0,55±0,51(1,0)	0,90±0,70(1,0)	0.043*
R4	0,87±0,72(1,0)	1,39±0,715(2,0)	0.007*
R5	0,10±0,30(0,0)	0,52±0,57(0,0)	0.001*
R6	0,19±0,40(0,0)	0,97±0,71(1,0)	0.001*

[d] Mann Whitney U test: values are given as mean ± standard deviation (median)

*p<0.05 statistically significant difference between groups

**Tablo S2. Detailed Regional Comparison of VCV in-group LUS Scores between Preoperative and Postoperative Measurements**			
	Preoperative (n=31)	Postoperative (n=31)	p
L1	0,26±0,51(0,0)	0,52±0,57(0,0)	0.043*
L2	0,35±0,55(0,0)	0,77±0,62(1,0)	0.006*

L3	0,32±0,475(0,0)	0,71±0,59(1,0)	0.008*
L4	0,71±0,59(1,0)	1,0±0,73(1,0)	0.114
L5	0,06±0,25(0,0)	0,48±0,57(0,0)	0.001*
L6	0,26±0,73(0,0)	0,97±0,795(1,0)	0.001*
R1	0,23±0,425(0,0)	0,58±0,67(0,0)	0.024*
R2	0,39±0,615(0,0)	1,39±0,67(1,0)	0.001*
R3	0,39±0,56(0,0)	0,94±0,57(1,0)	0.001*
R4	0,74±0,575(1,0)	1,55±0,77(2,0)	0.001*
R5	0,03±0,18(0,0)	0,71±0,59(1,0)	0.001*
R6	0,13±0,43(0,0)	1,19±0,70(1,0)	0.001*

[d] Mann Whitney U test: values are given as mean ± standard deviation (median)

*p<0.05 Gruplar arası istatistiksel olarak anlamlı fark
